# Design and Development of an Automated Pipeline for Medical Hyperspectral Image Acquisition, Processing, and Fusion

**DOI:** 10.3390/jimaging12030099

**Published:** 2026-02-25

**Authors:** Felix Wühler, Tim Markus Häußermann, Alessa Rache, Björn van Marwick, Carmen Wängler, Julian Reichwald, Matthias Rädle

**Affiliations:** 1 CeMOS—Research and Transfer Center, Technical University of Applied Sciences Mannheim, Paul-Wittsack-Straße 10, 68163 Mannheim, Germany; 2 Biomedical Chemistry, Clinic of Radiology and Nuclear Medicine, Medical Faculty, Mannheim of Heidelberg University, Theodor-Kutzer-Ufer 1-3, 68167 Mannheim, Germany

**Keywords:** hyperspectral imaging, multimodal data fusion, automated image processing, medical image analysis, unsupervised clustering

## Abstract

Automated and comprehensive processing of hyperspectral image data is increasingly important in academic research and medical technology. This study presents an automated processing pipeline that integrates hyperspectral image acquisition, analysis, multimodal fusion, and centralized data management to improve the interpretability of spectral information for biological tissue analysis. The pipeline supports modular hyperspectral data processing, fusion of complementary wavelength ranges, and scalable data storage, and was implemented in Python 3.13.3. The pipeline was evaluated using hyperspectral imaging data acquired from a coronal mouse brain section. Clustering-based analysis and spectral correlation metrics were applied to assess the impact of multimodal data fusion on spectral representation. Clustering of individual modalities yielded silhouette coefficients of 0.5879 for near-infrared data, 0.6020 for mid-infrared data, and 0.6715 for RGB data. Multimodal fusion reduced the silhouette coefficient to 0.5420 and enabled the identification of anatomical structures that were not distinguishable in any single modality. High spectral correlation coefficients exceeding 0.98 confirmed that spectral fidelity was preserved during fusion. These results demonstrate that automated multimodal hyperspectral data fusion can enhance the interpretability of biological tissue despite reduced clustering compactness. The proposed pipeline provides a structured framework for preclinical hyperspectral imaging workflows and supports exploratory biological analysis in medical imaging contexts.

## 1. Introduction

Hyperspectral Imaging (HSI) integrates optical spectroscopy and digital image processing to capture both spatial and spectral information across continuous bands of the electromagnetic spectrum [1]. In contrast to conventional panchromatic or trichromatic imaging, HSI offers a significantly higher spectral resolution, which allows for the precise identification of materials based on their unique spectral signatures [2,3]. This advanced capability enables its utilization across diverse sectors, including medical diagnostics, precision agriculture, environmental monitoring, and industrial quality control [2,4]. Particularly in medicine, HSI is gaining prominence for applications such as tissue characterization, cancer detection, dermatology, and ophthalmology [5].

The growing interest in HSI technologies is reflected in recent market forecasts predicting substantial growth. The global HSI market is expected to expand from approximately 847 million USD in 2024 to around 1.535 billion USD by 2029, reflecting a compound annual growth rate (CAGR) of 12.6% [4]. Specifically, the medical segment of the HSI market is anticipated to experience even stronger growth, reaching approximately 5.49 billion USD by 2032 with a CAGR of 13.6% from 2025 to 2032 [6]. Technological advancements that make HSI systems more affordable, portable, and user-friendly, alongside the integration of artificial intelligence (AI) and machine learning (ML) techniques for rapid and accurate data processing, are driving this significant market expansion [4,5].

Despite these promising developments, there remains a notable gap in research focusing on fully automated pipelines that seamlessly integrate data collection, processing, analysis, and storage of hyperspectral data [7,8]. Automated systems promise to significantly reduce manual labor, minimize errors, and enhance productivity, particularly in industrial and medical applications [7,9].

To address this gap, this paper introduces an automated pipeline designed for the collection, processing, analysis, and storage of hyperspectral data. Additionally, a method for merging complementary datasets acquired from different HSI systems is proposed, aiming to enhance the overall information content of the resulting datasets. To demonstrate the practical applicability of this approach, a pathological coronal section of a mouse brain is measured using multiple HSI systems and subsequently evaluated through the described automated pipeline. This experimental validation serves as a proof of concept, demonstrating technical feasibility and methodological integration, rather than providing a comprehensive biological performance assessment.

The manuscript is structured as follows: Section 1 elaborates on the potential of HSI, especially emphasizing medical applications, and underscores the necessity for automation. It also outlines the components and their interactions within the proposed pipeline. Section 2 provides detailed descriptions of the HSI data acquisition processes, preprocessing methods, and the techniques used for data fusion. Section 3 presents the outcomes and practical usability of the proposed methodology. Section 4 offers an in-depth discussion of the results and their broader implications, and finally, Section 5 concludes the paper by summarizing the key findings and suggesting future research directions.

### 1.1. Theory of Hyperspectral Image

**Figure 1:**
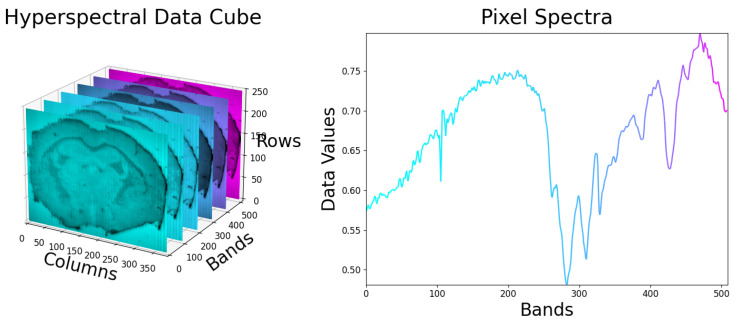
Example construction of a hyperspectral datacube.

HSI is a non-invasive imaging technique that captures and analyzes electromagnetic radiation interacting with a material. These interactions include transmission, reflection, emission, and, in certain cases, fluorescence and phosphorescence [10]. The electromagnetic spectrum is divided into various wavelength bands, encompassing frequencies that range from ultraviolet (UV) to visible light (VIS), infrared (IR), and beyond. By combining imaging with optical spectroscopy, HSI delivers both spatial and spectral information about a target [11]. Unlike conventional imaging methods such as panchromatic or trichromatic imaging, which offer limited spectral resolution, HSI divides the spectrum into multiple or hundreds of narrow, contiguous bands. The result is a stack of spectral images, called a datacube, described by its spatial resolution in x and y, and v or λ for the spectral resolution [3], shown in Figure 1.

A typical HSI system consists of three primary components: a light source, wavelength dispersion devices, and detectors [12]. The light source is used to illuminate or stimulate the material under analysis, with the choice of source, such as UV lamps, halogen lamps, or lasers, depending on the specific application [13]. Wavelength-dispersion devices, such as filters or spectrographs, are utilized for separating the broad-spectrum light into narrow, discrete wavelength bands for detailed analysis [13]. Detectors, including charge-coupled devices (CCDs), complementary metal–oxide–semiconductor (CMOS) sensors, indium-gallium-arsenide (InGaAs), Mercury–cadmium–telluride (MCT) focal-plane arrays (FPA), and photomultiplier tubes (PMTs), convert incoming photons into electrons and enable the quantitative mapping of spectral and molecular signatures [14,15,16].

**Figure 2:**
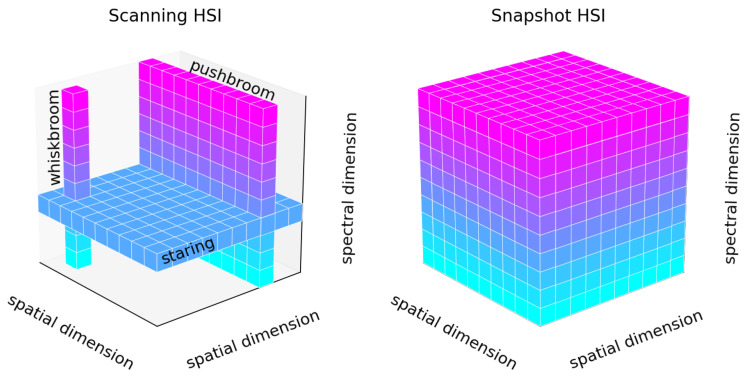
Hyperspectral image acquisition techniques.

Commonly, HSI acquisition techniques can be divided into point-scanning (whiskbroom), line-scanning (pushbroom), area-scanning (staring), and snapshot methods – shown in Figure 2. The scanning methods collect datacube information sequentially, capturing either individual pixel spectra or line spectra. In contrast, the snapshot method captures both spatial and spectral data simultaneously, using a mosaic sensor to collect multi-dimensional information [17].

### 1.2. Applications of HSI

HSI has found diverse applications due to its capacity to non-invasively acquire spatial and spectral information across a broad electromagnetic range. Key application domains include remote sensing, agriculture, pharmaceuticals, military surveillance, and medicine. In pharmaceutical quality control, UV-HSI is used to analyze active compounds in over-the-counter medications [18]. In military contexts, hyperspectral systems aid in long-range target detection and camouflage discrimination [19]. Given its broad analytical capability, HSI has gained traction in medicine as well. A custom multimodal microscope that integrates HSI, Raman spectroscopy, visible light spectroscopy (VIS), and NIR spectroscopy can discriminate between gray and white matter in a mouse brain [20]. In diabetic foot wound care, NIR-HSI supports ulcer healing prediction by mapping oxy- and deoxyhemoglobin distributions [21]. In oncology, HSI distinguishes between malignant and healthy tissue using spectral-fingerprint-based machine learning classifiers [22]. For head, neck, and laryngeal cancers, deep learning on 3D spatio-spectral data has achieved promising in vivo classification performance [23].

### 1.3. Processing and Analysis Overview

The variety of established and emerging methods for processing and analyzing HSI data are just as diverse as the acquisition methods. These methods differ in terms of their assumptions, complexity, and domain-specific suitability. Developing a broadly applicable solution remains challenging because processing methods and analysis strategies must be tailored to the characteristics and objectives of the data. Despite these challenges, an attempt was made to outline a generalized HSI processing and analysis workflow with a focus on medical applications.

#### 1.3.1. Preprocessing Overview

The initial step in data processing is the preprocessing of the HSI data to ensure quality and consistency, which allows for robust analysis. To obtain reliable spectra, acquisition-related artifacts such as instrumental noise, spectral spikes, and lighting irregularities must be mitigated to improve spectral quality and the signal-to-noise ratio [24]. Radiometric calibration converts raw detector intensities into meaningful radiance or reflectance values, compensating for systematic sensor and illumination effects. Subsequent normalization (e.g., min–max scaling or SNV) stabilizes the spectral scale, reducing residual intensity variability across pixels and samples [25]. Common approaches to spectral normalization include min–max scaling and SNV transformation, which stabilize the dynamic range and improve comparability across pixels and samples [26]. In multimodal and medical imaging settings, spatial alignment (registration) is an additional fundamental preprocessing operation. By mapping all images to a common coordinate system, registration establishes a spatial correspondence of anatomical and functional features, enabling joint analysis across modalities [27]. Overall, carefully designed preprocessing is a pivotal stage in modern analytical pipelines. Omitting these steps typically shifts the burden to more sophisticated, data-hungry network architectures that must learn to handle raw or noisy inputs. This is often at the expense of robustness and interpretability [28].

#### 1.3.2. Dimensionality Reduction

HSI data are characterized by a large number of spectral channels and features, resulting in extremely large datasets. A significant portion of this information is often redundant, giving rise to the well-known curse of dimensionality. This effect manifests as a decline in classification performance when only a limited number of training samples are available. Increasing dimensionality or the number of spectral bands exacerbates this challenge [29]. Dimensionality reduction in HSI can be achieved through feature extraction or feature (band) selection. Feature extraction transforms high-dimensional data into a lower-dimensional space while preserving or emphasizing information critical for class discrimination. However, this process can distort the spectral structure and yield features that are difficult to interpret. Common methods include Principal Component Analysis (PCA), which is frequently implemented through Singular Value Decomposition (SVD), Independent Component Analysis (ICA), or the Successive Projection Algorithm (SPA) [30]. In addition, non-linear manifold learning approaches such as Uniform Manifold Approximation and Projection (UMAP) are widely used for hyperspectral data visualization and exploratory analysis. More recent methods apply deep feature extraction via autoencoders or convolutional layers that learn complex patterns directly from raw datacubes [31]. Feature selection identifies a subset of representative spectral bands from the original data, maintaining the spectra’s physical interpretability [32].

#### 1.3.3. Data Analysis Approaches

Following dimension reduction, the data is grouped into meaningful categories using either traditional or deep learning models [33,34]. Classical machine learning classifiers, such as Support Vector Machines (SVMs), k-Nearest Neighbors (k-NNs), and Random Forests (RFs), remain popular for their robustness and interpretability. In scenarios with limited training samples, unsupervised clustering techniques such as k-means, hierarchical clustering, or Gaussian Mixture Models are often employed to group similar spectral signatures and assist downstream classification tasks [35,36]. However, deep learning architectures are increasingly used, including Convolutional Neural Networks (CNNs), stacked autoencoders, and hybrid models like convolutional neural network–graph neural network (CNN–GNN), which model spectral and spatial dependencies [37,38]. Also, recent trends focus on reducing the need for extensive labeled data through semi-supervised or self-supervised learning approaches [39,40].

#### 1.3.4. Post-Processing

Some workflows include an optional post-processing stage that refines the model output by enforcing spatial consistency and anatomical plausibility. Simple post-processing operations, including neighborhood-based filtering, spatial regularization, or morphological refinement, are commonly applied to suppress isolated misclassifications and produce spatially coherent tissue maps [22].

### 1.4. Data Image Fusion and Super-Resolution

Image fusion, defined as the integration of multiple complementary datacubes into a unified representation [41], is a key strategy for improving the informational content of hyperspectral imagery. A frequently discussed use case is hyperspectral super-resolution, in which low-spatial, high-spectral-resolution HSI is combined with high-spatial, low-spectral-resolution panchromatic or multispectral imagery to mitigate the inherent trade-off between spectral and spatial resolution [42,43]. These approaches explicitly design the fusion process to recover fine spatial detail while preserving or reconstructing rich spectral signatures. However, the present study does not address super-resolution in this strict sense. Instead, it adopts concepts from the fusion literature to combine multiple HSI systems with complementary spectral ranges and an RGB modality into a common representation. The primary goal is to improve material discrimination and tissue segmentation rather than spatial resolution.

In general, image fusion methodologies can be grouped into three categories: pixel-level, feature-level, and decision-level fusion [41,44,45]. Pixel-level fusion operates directly on the co-registered intensity values of input datacubes. Feature-level fusion combines higher-level representations derived from images. Decision-level fusion integrates the outputs of independent processing chains according to predefined inference rules or confidence measures [41,46]. Regardless of the chosen level, successful fusion requires that the input data be accurately aligned spatially, radiometrically, and semantically, so that corresponding structures are consistently represented in the fused output and can be reliably exploited by subsequent analysis steps [41]. In this work, we focus on pixel-level fusion of NIR, MIR, and RGB data as a prerequisite for downstream unsupervised clustering and anatomical interpretation.

### 1.5. Pipelines for Processing Hyperspectral Image Data

The increasing complexity and growing volume of hyperspectral data require a fully automated, modular pipeline that seamlessly integrates the acquisition, preprocessing, analysis, and storage phases [47,48]. Manual workflows not only lead to bottlenecks and inconsistencies but also limit scalability and reproducibility [48]. Industrial applications have demonstrated that throughput and reliability can be significantly improved by adopting end-to-end processing systems [49]. Despite the proliferation of hyperspectral platforms, few studies present comprehensive software architectures for the unified processing of diverse datasets [7,48], highlighting a clear research gap. By integrating different tools into a configurable pipeline, researchers and practitioners can shorten turnaround times, minimize human intervention, and ensure consistent data provenance, thereby enabling scalable, automated, and reproducible HSI workflows in both academic and applied settings.

### 1.6. Study Goal

The goal of this study is to introduce the concept of an automated pipeline for hyperspectral image data processing, with a specific focus on medical imaging applications. Additionally, the research aims to integrate multiple hyperspectral acquisition systems to enhance data diversity through an algorithm that merges datasets with complementary spectral information. The study includes the conceptual design and implementation of this pipeline in Python, creating a modular processing environment, integrating it with a central database for efficient data management, and incorporating a feedback loop to automatically retrieve and merge data from the repository. The pipeline’s functionality is validated by hyperspectral analysis of a mouse brain, demonstrating its ability to fuse spectral data and enhance biological insights. Ultimately, this research advances HSI by streamlining data acquisition, processing, and fusion, providing a scalable and universal solution for medical imaging applications.

## 2. Materials and Methods

This section provides a structured overview of the experimental workflow. It starts with specimen preparation and explains data acquisition and pipeline architecture. Followed by data processing, management, and validation.

### 2.1. Animal Specimens

The mouse line C57BL/6 was chosen on the basis of its broad application in neuroscientific research. In particular, the C57BL/6 mouse is often used as a standard model to study brain structures and cognitive functions, as it has stable genetics and well-characterised neuronal morphology, providing a reliable basis for segmentation and cluster analysis. As a standardized anatomical reference for the anatomical validation of the segmented pathological sections, the Allen Mouse Brain Atlas (P56, Coronal, Image 69/132) was used, enabling precise delineation of subregions in the C57BL/6 mouse brain [50]. For the sample preparation, a single biological replicate was cut to a thickness of 10 μm using a CM1950 cryostat (Leica Biosystems Nussloch GmbH, Nussloch, Germany). Before and after sectioning, the organ was stored at −80 °C to minimize tissue degradation. Although this study focuses on method development and validation of a multimodal hyperspectral processing workflow and therefore relies on a representative section from one biological replicate, the basic data processing has been established and validated in several independent studies from our group on murine brain and liver tissue [20,51,52].

### 2.2. Data Acquisition

**Figure 3:**
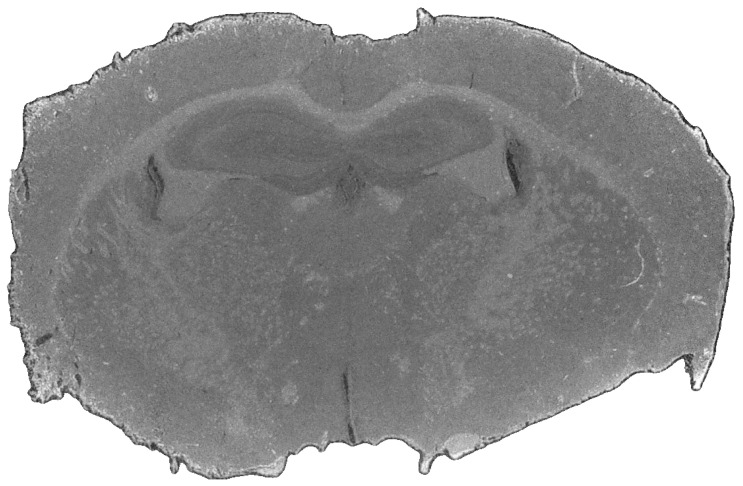
Mouse brain slice after with preprocessing, scanned with a Keyence VHX-7000 microscope (Keyence Deutschland GmbH, Neu-Isenburg, Germany) at 20× magnification.

For this study, the pipeline was evaluated using two in-house-developed scanning systems, a near-infrared (NIR) scanner [20] and a laser-based mid-infrared (MIR) scanner [51], representing complementary spectral ranges. Fourier transform infrared (FTIR) imaging, while commonly used as a standard approach in biomedical MIR analysis, was not considered due to its comparatively long acquisition times [51]. Additionally, an RGB reference image (Figure 3) was acquired using a Keyence VHX-7000 microscope at 20× magnification. The RGB modality was limited to a single-shot acquisition even though the system supported image stitching.

Each acquisition method exhibits differences in radiometric scales and spatial depth due to system-specific resolutions and detector configurations, shown in Table 1. These differences in scanning methods require robust and customized processing and alignment methods for data fusion. Therefore, they provide a good example of processing complementary hyperspectral datasets using the presented pipeline.

### 2.3. Pipeline Architecture

**Figure 4:**
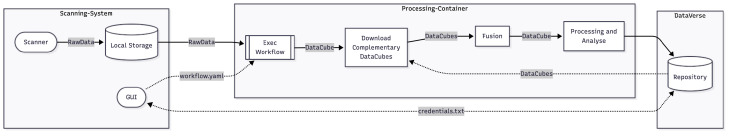
End-to-end dataflow of the pipeline, illustrating the automated process from local acquisition monitoring and GUI-based dataset assignment to containerized processing, data fusion, and synchronized upload to the Dataverse repository.

The proposed architecture, shown in Figure 4, integrates acquisition, processing, and storage of hyperspectral image data into an automated pipeline for biomedical imaging applications. Implemented entirely in Python, the system links multimodal spectral scanners, a modular processing environment based on the in-house-developed hsi-wizard v.0.1.42 [53], and a centralized research data repository (Dataverse v6.5). The complete system is containerized via Docker, deployed on a central compute server, and communicates through a Flask-based RESTful API. This approach ensures platform independence and facilitates future integration into distributed computing environments. By combining automation and modular design, the pipeline provides a reproducible, extensible, and fully traceable framework for processing and analysing hyperspectral biomedical data.

The acquisition stage interfaces with the scanners. A watchdog service continuously monitors the local file systems for new acquisitions and triggers automated processing via the pipeline, followed by upload to the Dataverse. This event-driven orchestration minimizes user interaction, thereby enabling continuous and autonomous data processing.

Additionally, a graphical user interface (GUI) has been implemented on each scanning system using the Python tkinter toolkit. This GUI facilitates assigning newly acquired data to specific Dataverse repositories during or immediately after scanning. Through the GUI, users can select target repositories and provide the corresponding workflow.yaml files, and update credentials.txt, thereby defining the processing workflow and metadata linkage for subsequent automated analysis. The GUI ensures that data are properly linked to their respective metadata and workflows during acquisition, maintaining consistent, structured data management across all systems.

Within the processing pipeline, each processing step is dynamically configured through the YAML-based workflow definition (workflow.yaml), which specifies the execution order and parameterization. Together with the auxiliary file (credentials.txt), containing access keys and metadata references for the Dataverse, this configuration defines both the analytical workflow and data management context. All results, including processed datacubes, segmentation maps, and metadata, are automatically uploaded to the Dataverse to ensure transparency and reproducibility.

To ensure robust data management, the processing container has an automated verification mechanism that checks whether newly captured records already exist in the target Dataverse repository. After successful data transfer from the acquisition system to the containerised processing environment, the Dataverse repository is queried using the metadata provided in credentials.txt. If supplementary data records are detected, an automatic merge process is initiated. The updated data set is then synchronised with the Dataverse repository, ensuring consistency, integrity, and reproducibility across the entire processing pipeline.

### 2.4. Data Processing

All raw datasets are transformed into standardized hyperspectral datacubes that capture both spatial and spectral dimensions. These datacubes contain embedded metadata such as acquisition parameters, wavelength ranges, and resolution information, ensuring reproducibility and traceability throughout the workflow. Importantly, the original raw, unprocessed acquisitions are preserved unchanged and stored alongside the processed datasets, enabling full reprocessing and reproducibility. An overview of the processing methods used for each dataset is provided in Table 2.

#### 2.4.1. Preprocessing

The preprocessing pipeline was tailored to the specific characteristics of each modality to correct modality-dependent artefacts and establish a radiometrically consistent basis for the subsequent registration and fusion. As shown in Table 2, the NIR data preprocessing included an initial spectral reference correction to compensate for illumination and detector fluctuations, implemented as a relative reflectance transform that converted raw intensities I(λ) to $R\left(\lambda \right)=I\left(\lambda \right)/I_{0}\left(\lambda \right)$ using a wavelength-matched reference spectrum $I_{0}\left(\lambda \right)$. Spectral outliers were removed using a modified Z-score procedure (threshold = 0.125, window = 13) by replacing detected spectral spikes with the local mean of the sliding window, after which background removal was applied, preserving the full datacube for all downstream processing steps. The spectra were then Min–Max normalized and reduced to *k* = 20 low-collinearity bands via the SPA [30]. This is done to improve stability, reduce redundancies prior to the fusion, and minimize bias in data analysis. The MIR data underwent a Gaussian Blur vignette correction (sigma = 10, epsilon = 5), followed by background removal and Min–Max normalization. For the RGB acquisition, vignette correction (sigma = 100, epsilon = 5) and background removal were likewise performed, and the intensities were normalized to align the radiometric scale with the hyperspectral modalities. Through these modality-adapted preprocessing steps, all datasets were transformed into a harmonized radiometric state, enabling accurate geometric registration and reliable multimodal data fusion.

#### 2.4.2. Resizing

Prior to the alignment process, the NIR and RGB data underwent a resizing procedure to match the spatial dimensions of the MIR datacube. This ensured a consistent, pixel-wise-aligned representation across all modalities. This process is an unavoidable trade-off. On the one hand, spatial interpolation inevitably reduces native spatial detail; on the other hand, it generates interpolated data. For the purposes of this research, all data has been scaled up for the sake of analysis.

#### 2.4.3. Registration

Geometric correction was performed to ensure spatial correspondence across modalities with inherently different sensor geometries, spatial resolutions, and acquisition conditions. Due to the area scanning data acquisition technology of the MIR scanner, the MIR dataset required intra-dataset layer registration to compensate for minor inter-frame shifts, which was performed using OpenCV’s Oriented FAST and Rotated BRIEF (ORB) feature detection alignment routines. Cross-modal registration relied on an Enhanced Correlation Coefficient (ECC)-based alignment [54]. This procedure enabled robust compensation for local and global spatial distortions introduced by differing optics, scanning mechanics, and thermal drift. These steps collectively produced a geometrically coherent multispectral dataset suitable for accurate spectral fusion and downstream analysis.

#### 2.4.4. Data Fusion

The multimodal fusion stage integrated the spatially registered NIR, MIR, and RGB data into a single hyperspectral cube. After geometric alignment, the spectral information from each modality was concatenated along the spectral dimension to preserve their complementary information. To maintain radiometric consistency across the fused dataset, post-merge normalization was applied to harmonize scale differences originating from heterogeneous sensor technologies. Then, the wavelengths for each input modality were reassigned to create a continuous, modality-aware spectral index enabling subsequent interpretation and algorithmic processing. This process yielded a high-dimensional multispectral-hyperspectral composite cube that captures the spectral richness of NIR and MIR while preserving the structural detail inherent in RGB imaging. This enhances the representational capacity of the final dataset.

#### 2.4.5. Clustering and Analysis

Unsupervised clustering was performed on the merged dataset to evaluate the total information content of all modalities and derive meaningful spatial segmentations. Mini-Batch KMeans (*k* = 9) was selected for its computational efficiency and scalability in high-dimensional fused hyperspectral feature spaces, serving as a simple and reproducible unsupervised baseline. Compared to more computationally demanding methods such as Gaussian Mixture Models or spectral clustering, Mini-Batch KMeans enables efficient clustering of large hyperspectral datasets while maintaining competitive performance. The algorithm was applied to the fused hyperspectral dataset with an extended iteration budget to ensure convergence. Clustering was executed uniformly across modalities, enabling direct comparison of segmentation performance between individual NIR, MIR, and RGB datasets and their fused counterpart. The resulting cluster maps offer an interpretable representation of the underlying tissue structures and serve as a quantitative reference for evaluating the advantages of the multimodal fusion strategy.

### 2.5. Data Management and FAIR Storage

The final stage of the automated pipeline is data storage, which is implemented via a local Dataverse instance. Dataverse is an open-source platform for publishing, storing, and citing research data originating from the Dataverse Project [55]. It ensures findable, accessible, interoperable, and reusable (FAIR) data management [55,56]. Within Dataverse, repositories (“Dataverses”) contain datasets, which in turn store data files and extensive metadata. In this pipeline, Dataverse provides a centralized, dedicated repository for all data generated throughout the workflow.

### 2.6. Validation Metrics and Performance Evaluation

As described in the previous section, the processing pipeline was executed once for each modality (NIR, MIR, and RGB) and once on the concatenated datacube with identical parameters to evaluate the added value of the fused data. This design enabled a direct comparison of information content and clustering performance between the unimodal datasets and the fused representation. The quality of spatial registration between the modalities was quantified using mutual information (MI), which measures the statistical dependence between the corresponding images and reflects how well structural information is preserved across modalities. Spectral similarities were quantified using the spectral correlation coefficient (SCC), which measures linear correlation between spectral signatures. This provides a direct assessment of consistency in spectral responses across modalities and within the fused representation. To evaluate the quality of the unsupervised segmentation, the mean silhouette coefficient across all pixels was computed. The silhouette coefficient summarizes how well each pixel fits within its assigned cluster compared to neighboring clusters. To objectively validate the classification, nine anatomically defined brain regions were selected using the Allen Mouse Brain Reference Atlas [50]. These can be divided into the main categories of grey matter (hippocampus, thalamus, and hypothalamus), white matter (corpus callosum, columns of the fornix, fimbria, and stria medullaris thalami), and cerebrospinal fluid (CSF) (lateral and third ventricles) spaces. Regions of gray matter are characterized by high cellularity and vascularization, producing spectra dominated by aqueous and protein-rich components. White matter tracts consist primarily of myelinated axons and therefore exhibit more lipid-rich spectral profiles. CSF compartments contain almost exclusively cerebrospinal fluid, resulting in spectra dominated by water absorption with minimal lipid or hemoglobin contributions.

## 3. Results

The automated processing pipeline produced standardized, radiometrically consistent, and spatially registered datacubes for the NIR, MIR, RGB, and fused datasets. The following section reports the segmentation results for each modality, followed by the outcomes of data fusion. Stability across 20 independent runs per modality is reported in Appendix A (Table A1).

### 3.1. NIR Processing Results

After undergoing modality-specific preprocessing, which included spectral reference correction, spike removal, background removal, min–max normalization, and SPA-based band reduction to *k* = 20, the NIR datacube produced a stable clustering solution with a mean silhouette coefficient of *s_NIR_* = 0.5879. This value indicates a coherent but moderately overlapping cluster arrangement.

**Figure 5:**
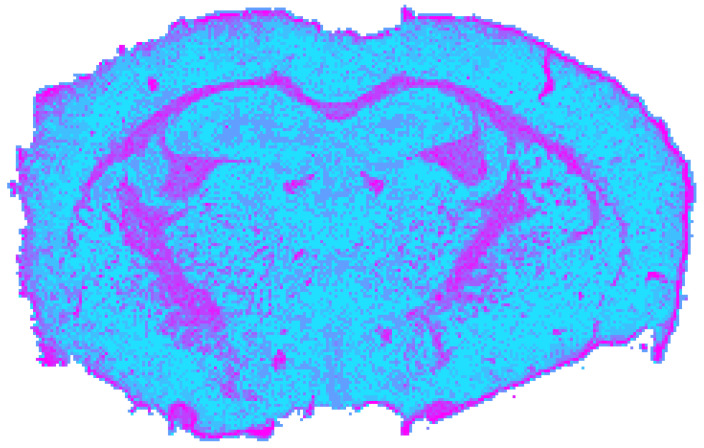
Spatial clustering map of the preprocessed NIR datacube obtained with Mini-Batch KMeans (*k* = 9), showing the unsupervised segmentation of the mouse brain section into spectral tissue classes.

Figure 5 shows that white-matter structures exhibited the strongest detectability. The corpus callosum, stria medullaris thalami, columnae fornicis, and fimbria hippocampi were all consistently identified as compact clusters. This reflects NIR reflectance sensitivity to myelinated tissue. Gray matter structures exhibited weaker contrast. The thalamus and hypothalamus were only partially separable, and the hippocampus remained undetected. CSF compartments were also not resolved. As shown in Table 3, the NIR clusters corresponding to white matter exhibit reduced water absorption and pronounced lipid-related CH overtone and combination bands. In contrast, clusters associated with gray matter and CSF are characterized by comparatively stronger water/protein absorption and weaker lipid contributions. Consequently, lipid-dominated clusters predominantly map to myelinated fiber tracts, while water- and protein-dominated clusters predominantly map to gray matter and fluid-filled compartments.

### 3.2. MIR Processing Results

Correcting the MIR dataset for vignetting and background prior to normalization yielded the most stable single-modality segmentation, with a silhouette coefficient of *s*_MIR_ = 0.6020. Compared to NIR, MIR data exhibited sharper cluster boundaries and stronger spectral discrimination.

**Figure 6:**
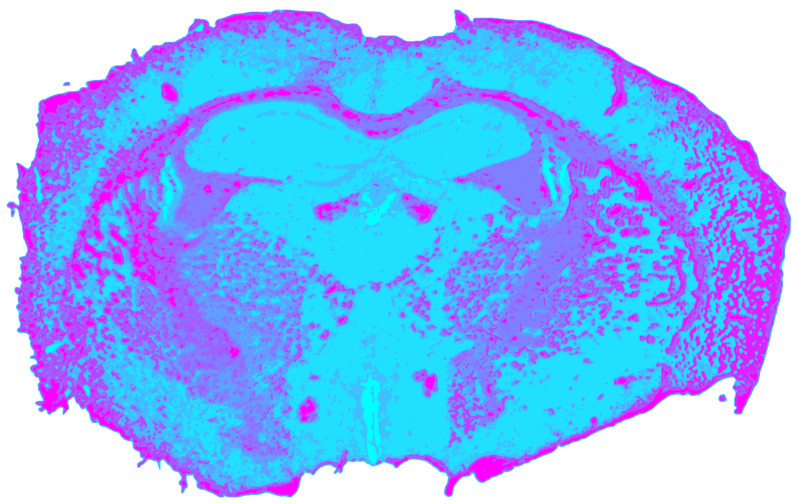
Spatial clustering map of the preprocessed MIR datacube obtained with Mini-Batch KMeans (*k* = 9), showing the unsupervised segmentation of the mouse brain section into spectral tissue classes. Colors are consistent across figures and represent categorical cluster labels.

As shown in Figure 6, gray matter regions were more clearly separated than in NIR, while major white matter tracts were consistently resolved as compact clusters. MIR imaging also provided the strongest contrast for CSF compartments, both the ventriculus lateralis and ventriculus tertius formed distinct clusters. As depicted in Table 3, this improved separability in the MIR domain reflects the dominance of lipid-associated CH_2_/CH_3_ stretching bands in white matter, stronger amide I/II and phosphate-related absorptions in gray matter, and water-dominated spectra in CSF, which together enhance interclass spectral contrast compared to the NIR range.

### 3.3. RGB Processing Results

**Figure 7:**
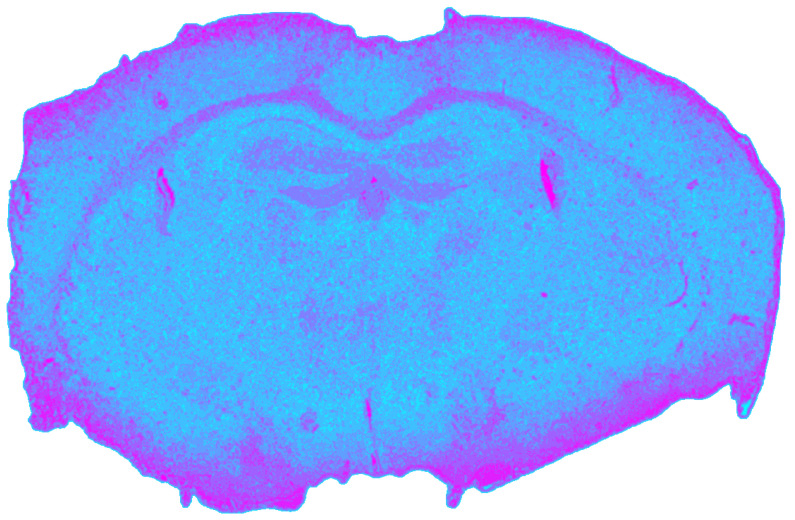
Spatial clustering map of the preprocessed RGB datacube obtained with Mini-Batch KMeans (*k* = 9), showing the unsupervised segmentation of the mouse brain section into spectral tissue classes. Colors are consistent across figures and represent categorical cluster labels.

Radiometrically normalized RGB images yielded a silhouette coefficient of *s*_RGB_ = 0.6715. However, this value likely overestimates the effective segmentation quality. Although the clustering (Figure 7) reproduced major cortical boundaries and superficial differences between gray and white matter, it provided the least chemically and anatomically specific separation among all single modalities. Unlike NIR or MIR data, RGB imaging lacks molecular absorption contrast and is therefore insensitive to lipid or protein composition. Instead, its discriminative power primarily arises from structural variations in tissue architecture. Consequently, the segmentation predominantly reflects macroscopic organization rather than biochemical heterogeneity. The ventriculus lateralis was the only CSF structure that was consistently outlined, while smaller or compositionally defined regions, such as the hippocampus or hypothalamus, remained undetected.

### 3.4. Fused-Data Processing Results

**Figure 8:**
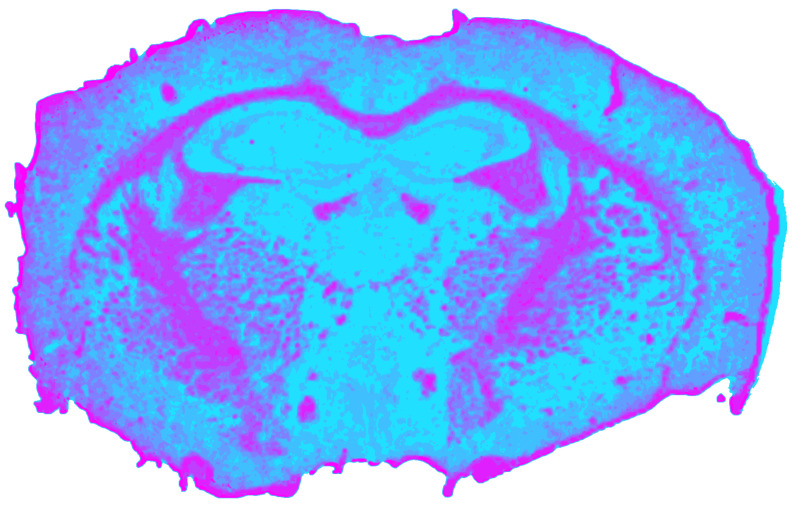
Spatial clustering map of the fused datacube obtained with Mini-Batch KMeans (*k* = 9), showing the unsupervised segmentation of the mouse brain section into spectral tissue classes. Colors are consistent across figures and represent categorical cluster labels.

The fused dataset (Figure 8) was constructed by geometric registration of all modalities, concatenation of their spectral signatures, and global post-fusion normalization. The resulting high-dimensional datacube supported a joint clustering analysis yielding a silhouette value of *s_fused_* = 0.5420, which is slightly lower than those of the single modalities, reflecting increased internal cluster variability in the expanded feature space. Despite the reduced silhouette scores, fused segmentation enhanced anatomical completeness. Grey matter structures that were previously undetected or weakly visible, such as the hippocampus, thalamus, and hypothalamus, were consistently segmented in the fused cube. All major white-matter tracts were preserved. CSF structures appeared with mixed quality: the ventriculus tertius and hypothalamus were clearly delineated, whereas the ventriculus lateralis showed reduced contrast compared to MIR or RGB alone.

Fidelity of the fusion process was evaluated using the spectral correlation coefficient (SCC), which quantifies the correlation between original modality spectra and their corresponding spectra within the fused cube. SCC values were SCC_NIR_ = 0.9999, SCC_MIR_ = 0.9899, SCC_RGB_ = 0.9999, indicating almost perfect preservation of modality-specific spectral characteristics after fusion. Mutual information (MI) between modalities further quantified cross-modal consistency. The fused dataset demonstrated markedly increased MI with all single modalities, most notably MI(NIR, Fused) = 1.5607, MI(MIR, Fused) = 1.0803, indicating that the fused cube captures complementary information unavailable to any single modality.

### 3.5. Comparison Between Single-Modality and Fused Results

**Figure 9:**
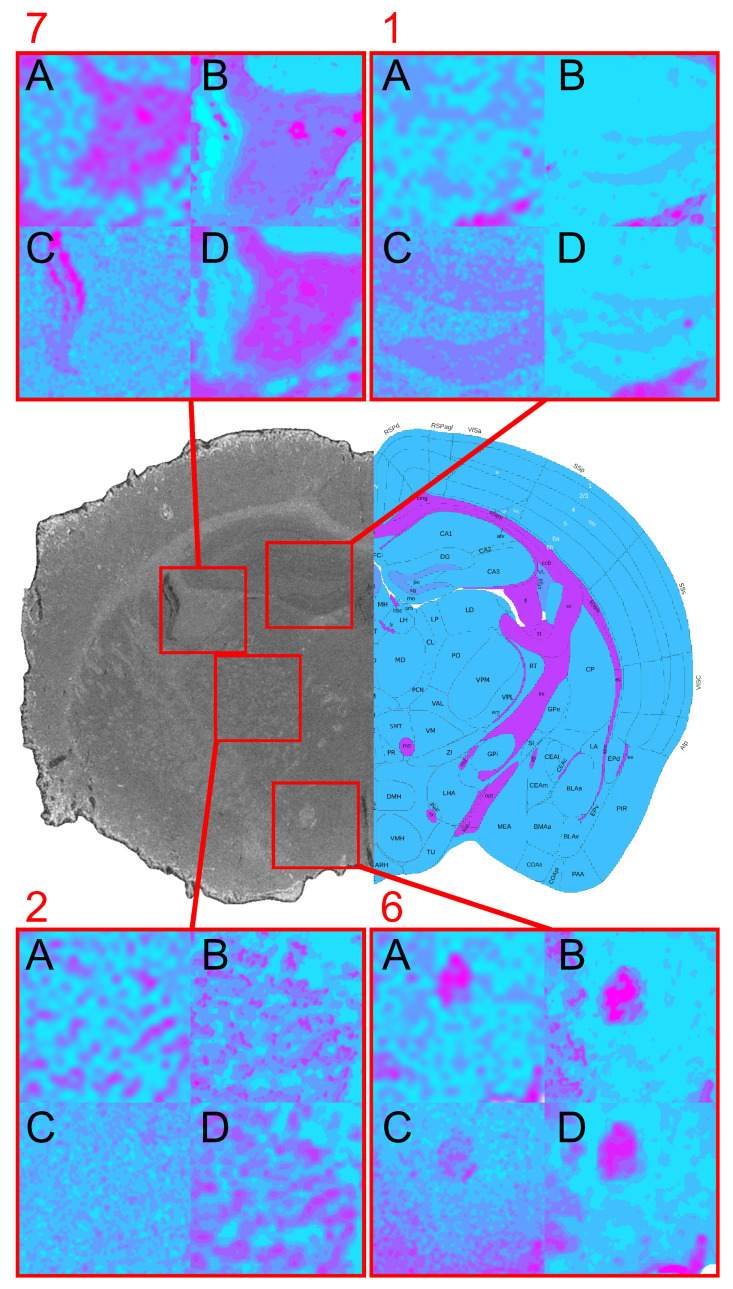
Region of interest comparison of the Mini-Batch KMeans datacubes to the Allen Brain Atlas reference. Showcasing the clusters of the Hippocampus (1), the Fimbria hippocampi (7), the Thalamus (2), and the Columnae fornicis (6) section for each processed modality (A: NIR, B: MIR, C: RGB, D: Fused).

Across the modalities, Figure 9, RGB yielded the highest silhouette coefficient but provided the least informative anatomical clustering, as the segmentation predominantly reflects superficial color gradients rather than true tissue contrast. MIR offered the most balanced and biologically meaningful single-modality performance, including reliable CSF detection, while NIR provided strong delineation of major white matter. The fused dataset combined these modality-specific strengths, producing the most anatomically complete segmentation despite the increased heterogeneity reflected in the silhouette metric. The structure-level comparison (Table 4) shows that the fused cube detected the largest set of anatomical regions across grey matter, white matter, and CSF compartments. Notably, structures undetectable in any single modality, such as the hippocampus, became identifiable only after multimodal integration.

## 4. Discussion

The presented work demonstrates that multimodal fusion compensates for modality-specific limitations, extending the anatomical completeness of tissue segmentation beyond what any single modality can provide. Although the fused cube yields slightly lower silhouette values due to increased internal variability, enhanced tissue discriminability, recovery of previously undetected structures, and high spectral correlation coefficients confirm that fusion preserves modality-specific signatures while capturing additional shared structures. The increased mutual information reflects the synergistic information gained by integrating NIR, MIR, and RGB data into a unified representation.

The quality of the fused dataset depends on the characteristics of the input modalities and the processing workflow. The automated pipeline reduces throughput times and improves reproducibility by using human-readable YAML workflows. This supports institutional digitalization efforts and makes spectral analysis more accessible. However, industrial deployment is limited by acquisition time and reliance on unsupervised algorithms, such as PCA and Mini-Batch KMeans clustering. Integrating deep learning could improve utility in supervised settings.

Methodologically, pixel-level fusion produced a more complete and biologically meaningful representation of the ex vivo mouse brain section, recovering major white matter and CSF compartments, as well as additional grey matter structures, including the hippocampus, that were absent in the unimodal results. The reduced silhouette coefficient is better understood as a consequence of increased heterogeneity in the fused feature space rather than degraded segmentation.

Interpretation of clustering quality is constrained by global metrics, a single unsupervised algorithm, and evaluation on one sample without specimen-matched histology. Future studies require larger cohorts, multiple brain levels, voxel-level validation, and alternative clustering approaches that leverage spatial context.

Beyond fusion, the automated, containerized, YAML-configurable pipeline tightly integrates acquisition, processing, and FAIR-compliant data storage. This ensures reproducibility and consistent parameterization across experiments. However, the system is still tailored to a specific hardware setup. Furthermore, the fusion requires resampling of NIR and RGB data to match the MIR shape. This process may introduce interpolation-induced smoothing, which can slightly reduce spatial precision when interpreting fine anatomical features. Therefore, expert oversight is still necessary to detect configuration errors or registration failures.

A further limitation lies in the restricted number of specimens included in this study. The evaluation is based on a single ex vivo brain section from one C57BL/6 mouse, which limits the generalizability of the findings. Biological variability, regional differences across brain levels, and potential specimen-specific artifacts cannot be assessed under these conditions. As a result, the reported detection rates and segmentation consistency should be interpreted as proof of concept rather than definitive performance estimates. Future work should incorporate larger cohorts and multiple anatomical levels to better quantify robustness, sensitivity, and specificity across individuals.

Taken together, the fusion strategy and pipeline architecture provide a promising foundation for HSI but should be regarded as a flexible framework. Advancing the approach will require more sophisticated fusion models, spatially aware analysis, adaptive workflows, and extensions to in vivo or clinical settings, where motion, physiological variability, and acquisition-time constraints introduce additional challenges.

## 5. Conclusions

This paper introduces an automated pipeline for acquiring, processing, analyzing, and storing HSI data. The pipeline integrates existing scanning systems, the hsi-wizard, and Dataverse into a cohesive system, minimizing human involvement. The framework enables non-programmers to specify and reproduce complex analysis workflows by configuring the hsi-wizard via YAML files. From a medical data processing perspective, this design addresses common issues such as fragmented tool chains, limited traceability of processing steps, and poor reusability of spectral datasets. Therefore, the combination of standardized metadata handling, automated logging, and FAIR-compliant storage in Dataverse facilitates longitudinal studies, multicenter data integration, and the secondary use of archived HSI data in medical technology research and development.

Additionally, a novel algorithm for fusing HSI data was implemented, enabling the radiometric and spatial alignment of complementary HSI data and their integration across wavelength ranges. Experimental validation was conducted to demonstrate the algorithm’s capability to produce fused datasets with enhanced information richness.

Future research could focus on extending the fusion algorithm and the pipeline, as well as on quality-of-life improvements. It could explore new registration techniques to improve robustness and integrate additional processing and analysis algorithms. Thereby, the pipeline’s performance, adaptability, and user experience can be ensured.

## Figures and Tables

**Table 1:** Overview of raw datasets used in the hyperspectral imaging pipeline.

Property	NIR	MIR	VHX-7000 (20×)
Image Size [px]	260 × 180	2000 × 1600	1150 × 1000
Spatial Resolution [μm]	76	10	16
Spectral resolution	260 bands (Δλ = 2.0 nm, 960–1478 nm)	3 discrete bands (2790, 2962, 3700 nm)	3 channels (RGB)
Acquisition Principle	Whiskbroom	Area Scanning	Snapshot

**Table 2:** Comprehensive overview of methods applied to individual and fused datasets.

Processing Step	NIR	MIR	RGB	Merged
Preprocessing				
Spectral Reference correction	✓	–	–	–
Spike removal	✓	–	–	–
Vignetting removal	–	✓	✓	–
Background removal	✓	✓	✓	✓
Min–Max normalization	✓	✓	✓	✓
SPA band selection	✓	–	–	–
Geometric Correction/Registration				
ORB layer registration	–	✓	–	–
ECC alignment	✓	–	✓	–
Resize	✓	–	✓	–
Fusion				
Min–Max normalization	–	–	–	✓
Cube concatenation	–	–	–	✓
Clustering/Analysis				
Mini-Batch KMeans clustering	✓	✓	✓	✓

**Table 3:** Overview of atlas-derived regions used for anatomical interpretation of the clustering results.

#	Region (Latin)	Function	Key Anatomical/ Biochemical Features
Grey Matter			
1.	Hippocampus	Memory formation	Cell body-rich; strong water and protein bands (NIR ∼ 1450 nm, MIR amide I/II), weak lipid signals
2.	Thalamus	Sensory relay	Mixed neuronal/glial content; moderate water absorption, weak CH_2_/CH_3_ lipid peaks
3.	Hypothalamus	Homeostasis, endocrine control	Vascularized; high water and protein signatures, weak lipid response in IR
White–Matter			
4.	Corpus callosum	Interhemispheric connectivity	Dense myelin; strong lipid CH bands (NIR ∼ 1700 nm, MIR 2850–2950 cm^−1^), low water
5.	Columnae fornicis	Limbic signal conduction	Myelinated tract; high lipid absorptions, minimal water/protein signals
6.	Fimbria fornicis	Hippocampal output	Compact myelin-rich bundle; dominant CH stretches, low aqueous content
7.	Stria medullaris thalami	Limbic–thalamic relay	Thin white matter; lipid-based spectrum, subdued water/protein presence
CSF Compartments			
8.	Ventriculus lateralis	CSF circulation	Water-dominated spectra (NIR ∼ 1450 nm, MIR ∼ 1640 cm^−1^); negligible lipid/protein
9.	Ventriculus tertius tertius	CSF passage	Similar water-dominant profile; IR signal lacks CH or amide peaks

**Table 4:** Objective detection quality across modalities for selected anatomical structures, grouped by tissue compartment.

#	Structure (Latin)	NIR	MIR	RGB	Merged
Grey Matter					
1.	Hippocampus	–	–	–	✓
2.	Thalamus	△	✓	–	✓
3.	Hypothalamus	△	△	–	✓
White Matter					
4.	Corpus callosum	✓	✓	△	✓
5.	Stria medullaris thalami	✓	✓	–	✓
6.	Columnae fornicis	✓	✓	–	✓
7.	Fimbria hippocampi	✓	✓	–	✓
CSF Compartments					
8.	Ventriculus lateralis	–	✓	✓	▽
9.	Ventriculus tertius	–	✓	–	△

Legend: ✓ = distinct anatomical delineation; △ = moderate detectability; ▽ = low detectability; – = no reliable structural visibility.

## Data Availability

The datasets generated and analyzed during the current study are not publicly available due to ethical and institutional restrictions related to animal research data but are available from the corresponding author upon reasonable request.

## Abbreviations

The following abbreviations are used in this manuscript:

BRIEF	Binary Robust Independent Elementary Features
CAGR	Compound Annual Growth Rate
CCD	Charge-Coupled Device
CMOS	Complementary Metal-Oxide-Semiconductor
CNN	Convolutional Neural Network
CNN-GNN	Convolutional Neural Network–Graph Neural Network
CSF	Cerebrospinal Fluid
ECC	Enhanced Correlation Coefficient
FAIR	Findable, Accessible, Interoperable, Reusable
FAST	Features from Accelerated Segment Test
FPA	Focal-Plane Arrays
GUI	Graphical User Interface
GNN	Graph Neural Network
HSI	Hyperspectral Imaging
ICA	Independent Component Analysis
InGaAs	Indium-Gallium-Arsenide
IR	Infrared
k-NN	k-Nearest Neighbors
MCT	Mercury-Cadmium-Telluride
MI	Mutual Information
MIR	Mid-Infrared
NIR	Near-Infrared
NIH	National Institutes of Health
ORB	Oriented FAST and Rotated BRIEF
PCA	Principal Component Analysis
PMT	Photomultiplier Tube
REST	Representational State Transfer
RF	Random Forest
SCC	Spectral Correlation Coefficient
SNV	Standard Normal Variate
SPA	Successive Projection Algorithm
SVM	Support Vector Machine
UV	Ultraviolet
VIS	Visible (Light)

## Appendix A. Stability Analysis of MiniBatch-KMeans Clustering

MiniBatch-KMeans was executed 20 times per modality using different random seeds. For each run, the mean silhouette coefficient was computed.

**Table A1:** Maximum and minimum mean silhouette coefficients across 20 independent runs with different random initializations per modality. Δ denotes the absolute difference.

Modality	Max Silhouette	Min Silhouette	Δ
NIR	0.6526	0.5829	0.0697
MIR	0.6148	0.6066	0.0082
RGB	0.6776	0.6681	0.0095
Fused	0.5442	0.5349	0.0093
